# Hospital-Based Perinatal Practices and Duration of Exclusive Breastfeeding in Mexican Mothers

**DOI:** 10.3390/children12081091

**Published:** 2025-08-20

**Authors:** Citlalli de los Ángeles Chávez-López, Clío Chávez-Palencia, Claudia Hunot-Alexander, Alfredo Larrosa-Haro, Anel Ibarra-Ortega, Sara Nayeli Acosta-Real, Edgar Manuel Vásquez-Garibay

**Affiliations:** 1 Centro Universitario de Ciencias de la Salud, Instituto de Nutrición Humana, Universidad de Guadalajara, Guadalajara 44340, Mexico; angeles.chavez.nutricion@gmail.com (C.d.l.Á.C.-L.); alfredo.larrosa@academicos.udg.mx (A.L.-H.); anel.ibarraortega@cw.bc.ca (A.I.-O.); edgar.vgaribay@academicos.udg.mx (E.M.V.-G.); 2 Centro Universitario de Tonalá, División de Ciencias de la Salud, Universidad de Guadalajara, Tonalá 45425, Mexico; 3 Centro Perinatal Ontekauani, Guadalajara 44692, Mexico; sara0187@hotmail.com

**Keywords:** breastfeeding promotion, exclusive breastfeeding, hospital maternity services, perinatal practices

## Abstract

**Highlights:**

**What are the main findings?**

- The use of human milk substitutes and bottles during hospitalization, as well as at discharge, was significantly associated with shorter exclusive breastfeeding duration.
- Delayed breastfeeding initiation, absence of continuous rooming-in, and being a first-time mother were significant predictors of not achieving six months of exclusive breastfeeding.

**What is the implication of the main finding?**

- The limited adherence to the Baby-Friendly Hospital Initiative practices in private hospitals may contribute to early cessation of exclusive breastfeeding, highlighting the need to strengthen institutional commitment and professional training in these settings.
- Practical and policy-level strategies should focus on improving breastfeeding support in maternity care, including implementing evidence-based guidelines, monitoring compliance, and providing continued postpartum support.

**Abstract:**

**Background/Objectives:** The initiation and maintenance of breastfeeding depend on internal and external factors that can either support or hinder its success. This study aimed to examine the association between hospital-based perinatal practices and the duration of exclusive breastfeeding among Mexican mothers of infants under one year of age. **Methods:** An analytical cross-sectional study was conducted in Guadalajara, Mexico, using a structured questionnaire developed in Google Forms and distributed via social media managed by healthcare professionals. Elegible participants were mothers of infants aged 6 to 12 months. Data were collected between March and November 2022 and included information on infant feeding at six months, sociodemographic and obstetric characteristics, breastfeeding education, hospital-based practices, and professional support during birth. A sample size of 323 participants was estimated on a 95% confidence level, 30% expected prevalence, and 5% margin of error. Statistical analyses included chi-square tests, odds ratios, Mann–Whitney U tests, and multivariate analyses. **Results:** A total of 326 mothers participated. Exclusive breastfeeding lasted less than six months for 63.5% of infants, while 36.5% were exclusively breastfed from birth to six months. Bottle use in the hospital, provision of human milk substitutes during the hospital stay, and at discharge were significantly associated with shorter exclusive breastfeeding duration (*p* < 0.001). Predictors of not achieving six months of exclusive breastfeeding included primiparity, delayed initiation beyond the first postpartum hour, and lack of continuous rooming-in. **Conclusions:** Hospital-based practices significantly influence exclusive breastfeeding duration. Strengthening maternity care policies may improve adherence to recommended feeding practices.

## 1. Introduction

According to the World Health Organization (WHO) and the United Nations Children’s Fund (UNICEF), human milk (HM) is the optimal food for infants, providing essential nutrients necessary for healthy development and growth. They recommend the initiation of breastfeeding (BF) during the first hour after birth, regardless of delivery type, exclusively breastfeeding (EBF) for the first six months and continuing BF alongside complementary foods up to two years of age or beyond [1].

Despite the multiple benefits of BF for both maternal and child health- including reduced risks of infections, chronic diseases, and maternal cancers— [2], only 44% of infants under six months of age are exclusively breastfed worldwide [1]. In Mexico, according to the National Health and Nutrition Survey (ENSANUT 2021–2022), only 33.6% of infants are exclusively human milk fed during the first five months [3]. This low rate is influenced by multiple factors, including cultural beliefs (e.g., breast milk insufficiency and the need to supplement with water and teas), social norms regarding public BF and duration, limited access to professional support, and the aggressive marketing practices for human milk substitutes (HMS) [4]. HMS are defined as any food marketed or otherwise represented as a partial or total replacement for human milk, whether suitable for that purpose or not [5].

Hospital-based perinatal practices experienced by mother–infant dyads can significantly influence a woman’s decision to breastfeed. For example, if a newborn whose mother intends to breastfeed exclusively receives HMS during the hospital stay, it may reduce the likelihood of continued EBF after discharge [6]. To protect, promote and support breastfeeding, the WHO and UNICEF developed the Baby-Friendly Hospital Initiative (BFHI) as a global program in 1991 [7]. This global program aims to support mothers in making informed feeding decisions, to encourage early initiation of BF, to promote EBF for the first six months, and eliminate the distribution or low-cost sale of HMS in maternity facilities [8,9].

Adherence to the ten steps of the BFHI has demonstrated positive effects on BF outcomes in the short, medium and long term. Several studies have shown a dose-response relationship between the number of BFHI steps implemented and the likelihood of successful initiation and duration of BF [2,10,11]. For instance, greater exposure to BFHI practices has been associated with higher rates of EBF and continued BF up to one year postpartum [12,13]. These benefits are observed not only during the immediate postnatal period but also in sustaining optimal feeding practices over time.

Avoiding the administration of foods or fluids other than HM to newborns is essential for establishing successful BF and minimizing early weaning. In addition to hospital-based practices, such as immediate BF initiation, uninterrupted skin-to-skin contact after birth, defined as placing the naked newborn prone on the mother’s bare chest immediately after delivery [14], rooming-in (the practice of keeping the mother and newborn together 24 h a day in the same room), and avoiding bottles or pacifiers, continued support after discharge remains crucial [14]. Step 10 of the BFHI emphasizes the importance of connecting families with community-based support systems. Community and peer support programs, such as mother-to-mother counseling or home visits, have been shown to play a vital role in sustaining BF practices beyond hospital settings [6,15]. Healthcare professionals are key actors in BF promotion, as their attitudes, counseling skills, and adherence to evidence-based guidelines can directly influence maternal confidence and BF duration [2,16,17].

By recruiting participants through online platforms, this study reached a population of mothers with internet access, reflecting an increasingly relevant demographic in Mexico. These findings help update current knowledge on EBF determinants in diverse populations.

Therefore, the main objective of the study was to examine the association between hospital-based perinatal practices and the duration of EBF among Mexican mothers of children under one year of age. Specifically, the study sought to answer the following questions: (1) What hospital-based perinatal practices are associated with the duration of exclusive breastfeeding? and (2) What are the sociodemographic characteristics of the mothers who participated in the survey?

## 2. Materials and Methods

### 2.1. Study Design and Participants

An analytical cross-sectional study was conducted between March and November 2022 in Guadalajara, Jalisco, Mexico. The study included healthy Mexican mothers aged 18 years or older who had healthy infants. Participants were required to reside in Mexico, have internet access and the use social media platforms, as the recruitment and data collection processes were conducted online. At the beginning of the virtual questionnaire, participants were presented with an informed consent form outlining the purpose of the study, the voluntary nature of their participation, and the confidentiality of their responses. Only those who provided consent were able to proceed to the full questionnaire.

Inclusion criteria for participation were (1) mothers with infants between 6 and 12 months of age; (2) mothers with full-term birth (between 37 and <42 weeks of gestation); (3) birth weight appropriate for gestational age (2.5 to 4.0 kg); (4) healthy mothers and infants; (5) internet access and the use of social media platforms; and (6) mothers had consented to participate voluntarily in the study. Mothers who had multiple pregnancies, preterm births, or any pathology that prevented adequate BF were not included. Infants who were hospitalized at birth in the neonatal intensive care unit or who had any genetic, congenital or chronic pathology were excluded. Women who answered the questionnaire incorrectly or incompletely were excluded.

The minimum required sample size (*n* = 323) was calculated by the following equation: *n* = [DEFF × Np(1 − p)]/[(d^2^/Z21 − α/2 × (N − 1) + p × (1 − p)] [18] and verified using Epi Info™ software version 3.01 [19]. Parameters included a total population size (N), an expected prevalence (p) of 30%, based on national data on EBF prevalence in Mexico, a confidence limit (d) of 5%, and a design effect (DEFF) of 1. A non-probabilistic snowball sampling method was used, where mothers were enrolled as they met the inclusion criteria.

### 2.2. Data Collection and Instrument

Two lactation advisors (C.d.l.Á.C.-L., and C.C.-P) and one International Board Certified Lactation Consultant (S.N.A.-R., IBCLC) collaboratively designed the instrument based on a comprehensive review of the scientific literature. Originally developed as part of the first author’s (C.d.l.Á.C.-L.) master’s thesis, the questionnaire was adapted for this study, selecting only the variables relevant to the present objectives. Content was aligned to address variables identified as relevant in the literature and national health surveys, ensuring contextual adequacy for the Mexican population.

The full instrument consisted of 82 items, five open-ended and 77 multiple-choice, distributed across ten thematic sections and was administered using Google Forms. However, only eight of these were included in the current analysis, excluding sections on family and social support, and psychological factors. The eight sections analyzed were: (1) general information (personal data, inclusion and exclusion criteria); (2) sociodemographic factors (age, place of residence, marital status, education level, family type); (3) socioeconomic factors (based on the socioeconomic level index); (4) gynecological and obstetric history (type and duration of pregnancy, parity); (5) breastfeeding education (timing, provider, perceived usefulness); (6) hospital-based practices (in line with the ten steps of the BFHI); (7) biological and physical barriers; and (8) breastfeeding duration.

To evaluate the clarity, wording, and overall comprehension of the items, a qualitative pilot test was conducted with 30 mothers to assess the clarity, wording, and comprehension of the questionnaire items. Feedback obtained during the pilot was used to refine question structure and language. Because the purpose was instrument refinement, no statistical analyses were performed on pilot data.

Survey dissemination was carried out via social media platforms, primarily Instagram and Facebook, using stories, posts, and direct messages. Healthcare professionals with an established maternal audience, including pediatricians, gynecologists, lactation advisors, and IBCLSs, shared the link to the questionnaire. Clinical personnel were not involved in data collection or analysis.

### 2.3. Variables and Classifications

The comparison groups were defined according to the duration of EBF: less than six months versus six months or more. The classification followed the WHO definition of EBF [20], in which the infant receives only HM—including expressed or donated milk—with no other liquids or solids, not even water. Exceptions include oral rehydration solution, and vitamin, mineral, or medication drops and syrups.

Predominant breastfeeding (PBF) refers to the infant receiving HM (expressed or donated) as the main source of nourishment, while also consuming water, water-based drinks, juices, oral rehydration salts, or supplements [21]. In this study, PBF was defined as feeding (directly or expressed) along with HM or other foods [21,22]. It was further categorized into three groups based on maternal perception: more than 50% HM, less than 50% HM, and equal proportions (50% HM/50% HMS). These estimates were based on the amount of HM received—either directly from the breast or expressed—compared to HMS intake [23]. Finally, HMS was defined as the use of substitutes in place of HM, with the infant receiving no HM at all [22].

In addition, sociodemographic, economic, gynecological-obstetric, and BF information and education factors, as well as hospital-based practices and care from health professionals at birth, were evaluated. The mother’s socioeconomic level was measured via the socioeconomic level index of the Mexican Association of Market Research and Public Opinion Agencies, which consists of six questions about the education of the head of the household, number of bedrooms, number of complete bathrooms, number of people employed, number of cars and internet access. According to the score obtained, the resulting score categorizes socioeconomic level as A/B (highest) to E (lowest) [24].

The general classification of family type was initially based on kinship criteria [25]; however, for the final analysis, it was operationalized into two categories: (1) simple or extended nuclear families, composed of a mother and father with their children; and (2) other family types, including single-parent households or families consisting of parents and additional related and/or unrelated individuals.

On the other hand, the Mexican states of residence of the mother and infant were classified into four regions [26]: (a) North (8 entities): Baja California, Baja California Sur, Coahuila, Chihuahua, Durango, Nuevo León, Sonora, Tamaulipas; (b) Center (12 entities): Aguascalientes, Colima, Guanajuato, Jalisco, Michoacán, Nayarit, Querétaro, Morelos, rest of the State of Mexico, San Luis Potosí, Sinaloa, Zacatecas; (c) Mexico City: Metropolitan Zone of the Valley of Mexico, encompassing Mexico City and suburban municipalities of the State of Mexico; and (d) South (11 entities): Campeche, Hidalgo, Chiapas, Guerrero, Oaxaca, Puebla, Tlaxcala, Quintana Roo, Tabasco, Veracruz, Yucatán.

Data were collected using a structured electronic questionnaire specifically developed for this study. The questionnaire was disseminated via social media platforms to reach the target population. It included items on participant identification, sociodemographic and socioeconomic characteristics, gynecological and obstetric history, prior BF education, hospital-based practices, and support received from health professionals.

### 2.4. Statistical Analysis

Kolmogorov-Smirnov test was used to assess the normality of quantitative variables. For qualitative variables, chi-square test was applied to identify factors associated with EBF. Odds ratios (ORs) with 95% confidence intervals (CIs) were calculated to estimate the likelihood of EBF among mothers exposed to specific risk factors compared to those unexposed. For non-normally distributed quantitative variables, Mann-Whitney U test was used to compare differences between mothers who practiced EBF and those who did not.

Finally, a multivariate binary logistic regression analysis was conducted using independent variables that showed significant associations with the dependent variable. Model performance was calculated using the Nagelkerke R^2^ to estimate explained variance and Cohen’s kappa coefficient to assess classification accuracy. The Hosmer-Lemeshow goodness-of-fit test was used to assess the model’s adequacy, with a *p*-value > 0.05 indicating acceptable fit. Statistical significance was set at a *p*-value < 0.05.

## 3. Results

### 3.1. Description of the Population

Data collection took place between March and November 2022. Of the 486 respondents, 160 were excluded for not meeting the selection criteria, resulting in a final sample of 326 participants.

The mean age of mothers was 30.7 ± 4.6 years (range: 18–49), and the mean age of the infants was 8.5 ± 2.1 months (range: 6–12). The median maternal age was 30 years (IQR 7) in the group with EBF duration of less than six months and 32 years (IQR 6) in the group with EBF duration of six months or more (*p* = 0.282). The median age of the infants was eight months (IQR 3), with no difference between the comparison groups (*p* = 0.585).

Table 1 presents the characteristics of the study population according to BF duration. Among all participants, 37.1% reported working as a professional or technician, whereas 24.5% identified as being housewives. With respect to mothers’ education, 61.7% of the participants reported having a bachelor’s degree, and 24.5% mentioned having a postgraduate degree. Most mothers were married (69.3%) or lived in a common law union (26.4%). Socioeconomically, over three quarters of the participants belonged to the highest income level. Responses were obtained from mothers residing in various Mexican states, with the central region accounting for 59.2% and the Mexico City region for 16.3%. Most mothers (81.3%) reported living in nuclear families (either simple or extended), while 18.7% belonged to other family types, including single-parent households or families composed of parents and additional related and/or unrelated individuals. The majority received care in private health facilities (80.4%), while a smaller proportion were attended in public health centers (19.6%).

### 3.2. Duration of Exclusive Breastfeeding

An analysis of all responses from the online questionnaire was conducted to assess the duration of the EBF. Participants were asked whether their infants were fed exclusively with HM from birth to six months. While 81.3% of mothers reported that their infants were exclusively breastfed at six months of age, only 36.5% maintained uninterrupted EBF throughout the first six months. This discrepancy can be explained by the introduction of HMS during the early postpartum period, particularly during the hospital stay, as reported in responses to the hospital-based practices section of the questionnaire. Although these infants later resumed EBF, likely supported by professional counseling or maternal determination, they no longer met the WHO criteria of continuous EBF from birth to six months.

When asked about the age at which any HMS were first introduced, 57.1% of participants reported offering them before the infant reached the first month of age, while 32.8% reported never offering HMS. In 3.4% of cases, HMS was introduced between one and five months, and in 6.7% of cases, between six and twelve months. Additionally, 2.5% of infants were introduced to HMS at seven months. Overall, 90.5% of HMS introductions occurred before the infant was five months old.

### 3.3. Gynecological-Obstetric Variables

Most participants were first-time mothers (73.9%), had a cesarean birth (72.4%), and 64.1% reported that their pregnancy was planned. On the other hand, being a first-time mother [*p* = 0.002; OR = 2.22; (95% CI 1.34–3.68)] and having a cesarean delivery [*p* < 0.001; OR = 2.49; (95% CI 1.51–4.09)] were risk factors for a shorter duration of EBF (Table 2).

### 3.4. Information and Prior Education on Breastfeeding

Many mothers had a history of BF. Only in the group of mothers with a BF duration of less than six months did 10% of them (*n* = 4) report no prior BF history; however, no significant associations were found. The duration of previous BF was equal to or greater than six months for 88% of the mothers. More than half of the women who had a history of previous pregnancies received BF education in the pregestational stage, with no significant differences between the groups regarding the duration of BF. Similarly, most women reported having received BF education during their last pregnancy. This education was provided in both the prenatal and postnatal stages for most mothers. We analyzed those who provided the most theoretical education and practical support on BF issues. Many participants received this education and practical support from certified BF advisors or consultants without finding significant associations. The degree of usefulness of the information and support received by mothers was not significantly associated with the duration of EBF (Table 2).

### 3.5. Hospital-Based Practices/Care and Support from Health Professionals

Analysis of hospital-based practices and professional support during the mothers’ hospital stay revealed that skin-to-skin contact within the first hour after birth was significantly more common among mothers who practiced EBF for six months or more (*p* < 0.001). In the same group, separation of the mother–infant dyad for more than one hour postpartum was less frequent (*p* < 0.001). These mothers were also more likely to initiate BF within the first hour after delivery (*p* < 0.001), experience 24-h rooming-in (*p* < 0.001), and breastfeed on demand (*p* < 0.001) (Table 3).

Notably, 55% of the mothers reported not receiving BF counseling from health personnel during their hospital stay. Among mothers who maintained EBF for six months or more, the in-hospital use of bottles was nearly absent (*p* < 0.001), and none reported giving HMS during hospitalization (*p* < 0.001). EBF was also the most common feeding practice at hospital discharge in this group (99.2%), compared to 51.7% in the group that did not maintained EBF (*p* < 0.001). In contrast, among those who discontinued EBF before six months, 41.5% reported partial BF, 3.4% predominant BF and 3.4% exclusive HMS use. These categories were grouped as “Other type of feeding” for analysis (Table 3).

Several hospital practices were identified as risk factors for shorter EBF duration: delayed skin-to-skin contact beyond the first hour after birth [OR = 2.75; 95% CI: 1.62–4.66], separation of the mother–infant dyad for more than one hour postpartum [OR = 4.52; 95% CI: 2.69–7.59], delayed initiation of BF [OR = 4.98; 95% CI: 2.99–8.31], and lack of 24-h rooming-in [OR = 13.16; 95% CI: 4.65–37.2] (Table 3). Some variables in Table 3 are marked as “ND” (not determined) because odds ratios could not be calculated based on the reported frequencies and percentages. This limitation was due to the low number of observations in one or both comparison groups for specific variables—particularly “Frequency of BF” and “Use of a bottle in the hospital”—which made statistical analysis unfeasible.

An analysis of the reasons for discontinuing EBF, based on questionnaire responses, showed that 51.8% of who initially reported not abandoning EBF (*n* = 272) had, in fact, introduced HMS during their hospital stay after delivery. Additionally, 2.6% discontinued EBF due to the initiation of complementary feeding, and 1.5% reported using HMS before the infant reached six months of age. However, when the reasons for abandoning EBF were analyzed dichotomously (HMS introduced during hospital stay vs. other reasons), no significant association was found with the duration of EBF (*p* = 0.697) (Table 4).

### 3.6. Variables Independently Associated with Duration of Breastfeeding for More than Six Months

A binary logistic regression model was performed to identify variables independently associated with maintaining EBF for six months or more. Three predictors remained significant in the final model.

Mothers who practiced 24-h rooming-in had higher odds of sustaining EBF (OR = 13.16; 95% CI: 4.65–37.2) compared to those who did not. Initiation of BF within the first hour postpartum was associated with increased odds of EBF at six months (OR = 4.98; 95% CI: 2.99–8.31). Additionally, having more than one child was a significant factor (OR = 2.22; 95% CI: 1.34–3.68), indicating that multiparous women were more likely to continue EBF (Table 5).

The model explained approximately 28.2% of the variance in EBF outcomes (Nagelkerke R^2^ = 0.282). Model fit was supported by the Hosmer–Lemeshow goodness-of-fit test (χ^2^ = 4.24; df = 4, *p* = 0.374). Classification agreement was measured with Cohen’s kappa coefficient value (κ = 0.402).

## 4. Discussion

This study identified key hospital-based perinatal practices and maternal characteristics associated with not achieving EBF for six months. While most mothers expressed the intention to breastfeed, only 36.5% of infants were exclusively breastfed with HM for the full six months, whereas 63.5% did not meet this recommendation. Early exposure to HMS, delayed initiation of BF, absence of rooming-in, and being a first-time mother were significantly associated with shorter EBF duration. Additionally, a lack of BF counseling and early HMS introduction—often within the first month—highlight missed opportunities for in-hospital support.

The EBF rate observed in this study is consistent with findings from ENSANUT 2021 and 2022, who reported an EBF prevalence of 33.6% [3]. However, as reported in the results, when asked about their infant’s feeding status at six months of age, 81% of mothers indicated they were practicing EBF. This significant increase in EBF at six months of age may reflect that most mothers aimed to feed their children with HM and received both theoretical and practical guidance on BF from specialists in the field. These factors may have encouraged mothers who had previously discontinued or interrupted EBF to resume BF and exclusively feed their children with HM by the time they reached six months of age. Nevertheless, 57.1% of HMS exposure occurred before one month of age, a critical period during which early introduction of HMS has been linked to a higher risk of early weaning [27].

Regarding sociodemographic factors, no significant associations were found between maternal age and the duration of EBF, which contrasts with the findings of Chimoriya et al. and Lechosa-Muñiz et al., who reported that older maternal age is associated with a higher likelihood of continued BF [28,29]. Similarly, maternal education, which was not notably high in this group of women, was not associated with EBF duration, in contrast to international studies showing higher maternal educational level is significantly associated with a longer BF duration [28,29,30,31].

A theoretical exercise was carried out by doubling the sample size, suggesting that the lack of associations may be attributable to a possible type II error due to the reduced sample size after data stratification. Most participants were engaged in paid employment, yet no association was observed between occupation and EBF duration. This contrasts with previous studies maternal employment have been linked to BF cessation [28,32,33]. Although many mothers were married or in a common-law union, marital status was not significantly associated with EBF duration. In contrast, other studies have reported that BF is practiced more frequently and for longer among married women compared to single women [30,34].

Fieldwork was conducted virtually during the COVID-19 pandemic, allowing participation from mothers residing in various states across Mexico. This may have introduced bias, as response frequencies and percentages were not evenly distributed across states. To address this, data were analyzed by region; however, no significant association was found between region and EBF duration. Additionally, most participants belonged to single or extended nuclear families, with no significant associations observed between family structure and EBF duration. This finding contrasts with that of Garfias-Barrera et al., who reported that belonging to an extended family was a risk factor for discontinuing BF [35].

Although no significant associations were found between EBF duration and the type of health center attended, most participants included in this research received care in private hospitals, with fewer treated in public health care centers. This differs from the findings of Niño et al. who reported that the public health services were associated with a longer BF duration [36]. Similarly, Unar-Munguía et al. reported that access to private health institutions was positively associated with EBF [37]. While several studies have reported that higher socioeconomic status is linked to a lower risk of BF cessation, most participants in this study belonged to the highest socioeconomic levels (A/B and C+), and no significant associations were observed with EBF duration [28,32,33]. Thus, a theoretical exercise doubling the sample size suggested that this lack of association may be due to a potential type II error related to the small sample size.

The number of children and the mode of birth of the infant were significantly associated with EBF duration. Being a first-time mother and having a cesarean birth were both associated with a shorter duration of EBF. These findings are consistent with previous studies [28,37,38]. It is well established that practices associated with cesarean delivery, along with the limited BF experience among first-time mothers, may negatively influence BF initiation and, consequently, its continuation.

Vural & Vural reported that, compared to prenatal education alone, combining prenatal sessions with individualized postpartum support increased EBF rates at six months [39]. However, the findings from the present study differ, as BF education during previous pregnancy(ies) or the most recent pregnancy, as well as the timing of that education, was not significantly associated with EBF duration. This difference could be explained by a probable type II error due to limited statistical power for this variable. Similarly, the history of BF and duration of previous BF were not significantly associated with the current EBF duration. This contrasts with findings by Wagner et al., who reported that previous BF experience (whether mothers had breastfed their previous children) was significantly associated with both BF initiation and duration [40]. Although many participants in this study received both theoretical education and practical support from certified BF advisors or consultants, no significant association was found with longer BF duration. This may be partly explained by the fact that in most cases (57%), participants interrupted EBF due to the introduction of HMS during their hospital stay after delivery.

One of the most relevant findings was the hospital-based practices and support from health professionals during the mother’s hospital stay at the time of delivery, most participants having been attended in private health care settings. BF frequency, use of a bottle, use of HMS during the hospital stay and the type of feeding at discharge, were all significantly associated with EBF duration. Although odds ratios could not be calculated for these variables, chi-square tests indicated significant association with EBF duration, which coincides with the findings from other studies [41,42].

Having first skin-to-skin contact after the first hour of birth, separation of the dyad for more than an hour postpartum, delayed initiation of BF, and lack of 24-h rooming, were all identified as risk factors for shorter EBF duration. These findings are consistent with previous studies and highlight the critical role of hospital-based practices in supporting EBF [41,43,44,45,46]. Notably, these factors align closely with the “Ten steps to successful BF” and the principles of the BFHI [8,16]. Although our questionnaire did not explicitly capture the reasons for mother–infant separation, it is plausible that such separations resulted from routine hospital protocols, postoperative recovery following cesarean birth, or institutional practices not fully aligned with BFHI guidelines. In Mexico, it is still common for private hospitals to separate mothers and infants for extended periods, particularly after surgical deliveries, which may delay the onset of lactogenesis II. This delay can lead to unsettled infant behavior and maternal concerns about insufficient milk production. In such contexts, both health professionals and parents may misinterpret normal newborn behavior as a sign of milk inadequacy, prompting the early introduction of HMS. This situation is further exacerbated by the aggressive marketing of HMS, which exploits parental anxieties with misleading claims, undermining maternal confidence and breastfeeding success [47]. Our findings emphasize the need to revise perinatal care protocols in private healthcare settings to prevent unnecessary separation and provide timely, evidence-based lactation support to foster successful BF. One of the most notable findings was that a high percentage of the participants (81%) reported not having abandoned EBF, while only 16.6% indicated discontinuation of BF due to reasons such as, maternal choice, or perceived low milk supply. However, an analysis of the questionnaire responses revealed that many of these mothers had, in fact, interrupted EBF through the introduction of HMS during their hospital stay, early initiation of complementary feeding, or the use of HMS at some point before six months of age of the infants. These findings coincide with those of McCoy & Heggie [41], who reported that HMS supplementation in breastfed infants negatively impacts BF duration.

To better understand the factors that promote the achievement of EBF for six months, several logistic regression models were constructed to identify associated variables. Being a first-time mother, initiating BF after the first hour postpartum and the absence of 24 h rooming-in during the hospital stay emerged as significant factors associated with not achieving EBF. These findings align with previous reports by other authors, underscoring the importance of addressing hospital-based practices in efforts to support EBF [38,45,48].

The main limitation of this study is its cross-sectional design, which inherently prevents the establishing of causal relationship between the identified variables and EBF duration. Additionally, recall bias may have affected participants responses, as the questionnaire relied on maternal self-report of past experiences during pregnancy, childbirth, and early postpartum. The use of a non-probabilistic snowball sampling method also limits the generalizability of the findings. While this strategy facilitated access to mothers with internet access who use social media, it may exclude those with limited digital connectivity or from rural or marginalized regions. Although participants were recruited from 29 of the 32 states of the Mexican states, demonstrating broad geographic coverage, the distribution of responses across states was uneven, which may affect sample representativeness. Furthermore, the use of an online questionnaire, although practical for wide dissemination, could have introduced errors or misunderstandings despite providing a contact number to resolve doubts. Finally, some variables(e.g., maternal education, socioeconomic status, BF history, and perceived usefulness of the practical support) may have been affected by type II error. Another potential limitation concerns the observed discrepancy between the proportion of mothers who reported EBF for six months and the proportions identified as having maintained EBF based on their reported practices. This divergence likely reflects a misunderstanding of the strict definition of EBF, particularly in relation to early supplementation with HMS during the hospital stay. While this may indicate a successful reestablishment of EBF after discharge, it also highlights the challenges of relying on self-reported data for accurately assessing adherence to EBF criteria and underscores the need to interpret maternal reports within the broader context of perinatal practices.

A key strength of this study is that, to our knowledge, few recent investigations in Mexico have explored factors associated with the EBF duration, from a multifactorial perspective in populations with higher educational and socioeconomic status and greater access to private health care. The study included mothers from various states across the Mexican Republic, most of whom belonged to higher socioeconomic levels. Despite receiving information and support from certified BF specialists, many participants were still exposed to suboptimal hospital-based practices that were strongly associated with shorter EBF duration. While the findings are not generalizable to the entire population, they provide valuable insights for future research, raise important new questions, and support the development of improved intervention strategies for BF promotion.

This study contributes novel insights by focusing on a population of predominantly higher-educated, higher-income Mexican mothers, many of whom received care in private healthcare settings. These characteristics distinguish our sample from those typically included in national BF studies, which often emphasize public hospital users or socially vulnerable populations. Despite the assumed advantages of personalized care in private institutions, our findings reveal that key BFHI practices were not consistently implemented. This suggests that gaps in hospital-based support for BF are not exclusive to public healthcare. By addressing an underrepresented group in the Mexican context, our study highlights the need to strengthen BFHI adherence across all sectors of the health system to ensure equitable breastfeeding support for all mothers.

To address the gaps identified, targeted interventions should be implemented in private healthcare settings. These include mandating training for health professionals on BF support, standardizing in-hospital practices such as early skin-to-skin contact, initiation of BF within the first hour, and uninterrupted 24-h rooming-in. A systematic review and meta-analysis by Habe et al. confirmed that implementation of the BFHI significantly improves both early initiation and EBF rates, underscoring the importance of consistent adherence to BFHI guidelines across settings [49]. Greater regulatory oversight is also needed to ensure that private institutions comply with national and international BF standards.

## 5. Conclusions

This study, conducted with participants from 29 of the 32 states of the Mexican Republic—primarily women with higher socioeconomic status and educational attainment who accessed private health care, provides valuable insights into the hospital-based factors influencing the duration of EBF. Despite their privileged access to health services and certified BF support, many mothers experienced suboptimal perinatal practices.

Being a first-time mother, delayed initiation of BF (beyond the first hour postpartum), delayed skin-to-skin contact, separation of the mother–infant dyad for more than one hour after birth, and lack of 24-h rooming-in were identified as risk factors for a shorter duration of EBF. Logistic regression analysis confirmed that being a first-time mother, starting BF after the first hour postpartum, and absence of shared accommodation during hospitalization were significant predictors of not achieving EBF for six months.

These findings highlight the need for policies requiring healthcare professionals to adhere to BFHI guidelines and the WHO Code, alongside peer support and supervision to improve EBF outcomes in private settings.

## Figures and Tables

**Table 1:** Frequencies and percentages of sociodemographic and economic variables of Mexican mothers (*n* = 326, Mexico, 2022).

Variables		Duration of EBF (*n* = 326)				*p* ^b^
		Less than Six Months (*n* = 207)		Greater than or Equal to Six Months *^a^* (*n* = 119)		
		*n*	(%)	*n*	(%)	
Mother’s occupation	Manager/Directive activities	21	(10.1)	22	(18.8)	0.310 *^c^*
	Professional and technical	87	(42)	34	(28.6)	
	Other	52	(25.1)	30	(25.2)	
	Housewife	47	(22.7)	33	(27.7)	
Mother’s education *^d^*	Higher education	183	(88.4)	98	(82.4)	0.127
	Basic and upper secondary education	24	(11.6)	21	(17.6)	
Marital status	Married or free union	197	(95.2)	115	(96.6)	0.529
	Single woman	10	(4.8)	4	(3.4)	
Region of residence	Northern Region	24	(11.6)	16	(13.4)	0.782
	Central Region *^e^*	120	(58)	73	(61.3)	
	Mexico City Region	36	(17.4)	17	(14.3)	
	Southern Region	27	(13)	13	(10.9)	
Socioeconomic level	High (A/B and C+)	171	(82.6)	90	(75.6)	0.129
	Middle and low (C, C−, D+ and D) *^f^*	36	(17.4)	29	(24.4)	
Family type	Simple or extended nuclear	164	(79.2)	101	(84.9)	0.208
	Other family types *^g^*	43	(20.8)	18	(15.1)	
Type of health center	Private	171	(82.6)	91	(76.5)	0.179
	Public	36	(17.4)	28	(23.5)	

EBF: Exclusive breastfeeding. *^a^* Responses from three participants (0.9%) reported that the duration of their EBF was seven months. *^b^* Mann-Whitney U or Chi-square test. *^c^* Housewife vs. paid job (official, manager and directive activities; professional and technician; other). *^d^* Higher education (Bachelor’s degree, specialty and postgraduate); Basic and upper secondary education (Primary, junior high school and high school). *^e^* Central region vs. other regions (North, Mexico City and South) *p* = 0.551. *^f^* Letters (A/B, C+, C, C−, D+, D) correspond to categories from the Mexican socioeconomic classification instrument. High level included A/B and C+, and middle and low levels included C, C−, D+, and D. *^g^* Including single-parent households or families consisting of parents and additional related and/or unrelated individuals. Note: Statistical significance was set at *p* < 0.05.

**Table 2:** Frequencies and percentages of gynecological-obstetric variables, history, duration and education on breastfeeding of Mexican mothers (*n* = 326, Mexico, 2022).

Variable		Duration of EBF (*n* = 326)				*p* ^b^	OR	IC 95%
		Less than Six Months (*n* = 207)		Greater than or Equal to Six Months *^a^* (*n* = 119)				
		*n*	(%)	*n*	(%)			
Number of children, n (%)	One child	165	(79.7)	76	(63.9)	0.002	2.22	1.34–3.68
	More than one child	42	(20.3)	43	(36.1)			
Way of birth	Cesarean section	164	(79.2)	72	(60.5)	<0.001	2.49	1.51–4.09
	Vaginal	43	(20.8)	47	(39.5)			
Pregnancy type	Planned	133	(64.3)	76	(63.9)	0.944	-	-
	Unplanned	74	(35.7)	43	(36.1)			
BF history *^c^* (*n* = 85)	Yes	36	(90)	45	(100)	0.097 *^d^*	-	-
	No	4	(10)	0	(0)			
Duration of previous BF *^e^* (*n* = 81)	<6 months	6	(16.7)	4	(8.9)	0.473 *^d^*	-	-
	Equal or >6 months	30	(83.3)	41	(91.1)			
History of BF education in previous pregnancy *^e^* (*n* = 85)	Yes	23	(57.5)	26	(57.8)	0.979	-	-
	No	17	(42.5)	19	(42.2)			
BF education in the mother’s last pregnancy	Yes	155	(75.6)	80	(66.1)	0.065	-	-
	No	50	(24.4)	41	(33.9)			
Time at which BF education was received *^f^* (*n* = 281)	Prenatal stage	36	(20.2)	15	(14.6)	0.481	-	-
	Postnatal stage	44	(24.7)	26	(25.2)			
	In both stages	98	(55.1)	62	(60.2)			
Person who provided the most theoretical education in BF *^g^* (*n* = 311)	Certified BM advisor or consultant	96	(48.7)	54	(47.4)	0.817	-	-
	Other *^h^*	101	(51.3)	60	(52.6)			
Usefulness of the theoretical information received *^g^* (*n* = 305)	Very useful or useful	173	(89.6)	107	(95.5)	0.700	-	-
	Moderately, little or not helpful	20	(10.4)	5	(4.5)			
Person who provided the most practical education in BF *^g^* (*n* = 286)	Health professionals or other *^i^*	73	(40.1)	37	(35.6)	0.448	-	-
	Certified BM advisor or consultant	109	(59.9)	67	(64.4)			
Usefulness of the practical information received *^g^* (*n* = 282)	Very useful or useful	160	(89.9)	99	(95.2)	0.116	-	-
	Moderately, little or not helpful	18	(10.1)	5	(4.8)			

BF: Breastfeeding. EBF: Exclusive breastfeeding. OR: odds ratio. CI: Confidence interval. *^a^* Responses from three participants (0.9%) reported that the duration of their EBF was seven months. *^b^* Chi-square test. *^c^* First-time mothers were excluded. *^d^* Chi-square test with continuity correction. *^e^* First-time mothers and those who reported that it was their first breastfeeding were excluded. *^f^* Mothers who reported not having received BF education were excluded. *^g^* Mothers who reported not having received theoretical or practical education in BF were excluded. *^h^* Health professionals: nurse, pediatrician, gynecologist, general practitioner, neonatologist, nutritionist; social networks, internet, books, family, support groups, doulas, support groups, I am a BF advisor/consultant, autonomous learning with support from different media. *^i^* Health professionals: nurses, pediatricians, gynecologists, general practitioners, neonatologists, nutritionists; family, friends, support groups, social networks, and BF advisors/consultants; independent learning with support from different media, midwives and doulas. Note: Statistical significance was set at *p* < 0.05.

**Table 3:** Hospital-based practices associated with the duration of exclusive breastfeeding in Mexican women (*n* = 326, Mexico, 2022).

Variable		Duration of EBF (*n* = 326)				*p* ^b^	OR	IC 95%
		Less than Six Months (*n* = 207)		Greater than or Equal to Six Months *^a^* (*n* = 119)				
		*n*	(%)	*n*	(%)			
Moment of first skin-to-skin contact	After the first hour following birth	85	(41.1)	24	(20.2)	<0.001	2.75	1.62–4.66
	During the first hour after birth	122	(58.9)	95	(79.8)			
Separation > 1 h after delivery	Yes	113	(54.6)	25	(21)	<0.001	4.52	2.69–7.59
	No	94	(45.4)	94	(79)			
Initiation of BF within the first hour after delivery	Yes	84	(40.6)	92	(77.3)	<0.001	4.98	2.99–8.31
	No	123	(59.4)	27	(22.7)			
24-h rooming-in	Yes	142	(68.6)	115	(96.6)	<0.001	13.16	4.65–37.2
	No	65	(31.4)	4	(3.4)			
Advice on BF during your hospital stay	Yes	95	(45.9)	50	(42)	0.498	-	-
	No	112	(54.1)	69	(58)			
Frequency of BF	On demand	161	(77.8)	119	(100)	<0.001	ND *^c^*	ND *^c^*
	With breaks or established schedules	46	(22.2)	0	(0)			
Using a bottle in the hospital	Yes	153	(73.9)	1	(0.8)	<0.001	*	*
	No	54	(26.1)	118	(99.2)			
Use of HMS in the hospital	Yes	186	(89.9)	0	(0)	<0.001	ND *^c^*	ND *^c^*
	No	21	(10.1)	119	(100)			
Type of feeding at hospital discharge	EBF	107	(51.7)	118	(99.2)	<0.001	*	*
	Other type *^d^*	100	(48.3)	1	(0.8)			

BF: Breastfeeding. EBF: Exclusive breastfeeding. HMS: Human milk substitute. OR: odds ratio. CI: Confidence interval. *^a^* Responses from three participants (0.9%) reported that the duration of EBF was seven months. *^b^* Chi-square test. *^c^* These values could not be determined on the basis of the frequencies and percentages reported. *^d^* Other type of feeding: predominant, partial or HMS. * Variables that reported very wide confidence intervals in the odds ratio test. Note: Statistical significance was set at *p* < 0.05.

**Table 4:** Reasons for abandoning exclusive breastfeeding based on maternal responses to the questionnaire (*n* = 326, Mexico, 2022).

Reason for Abandoning EBF	*n*	(%)
The baby was fed with some HMS during his hospital stay (after delivery) *^a^*	141	(43.3)
I did not abandon the EBF	119	(36.5)
By mother’s decision	14	(4.3)
Personal perception of low milk production	11	(3.4)
Start of complementary feeding	7	(2.1)
Rejection of the newborn or infant from the mother’s breast	6	(1.8)
Due to joining work/studies	4	(1.2)
Diagnosis of low milk production provided by a specialist	4	(1.2)
The baby was fed some HMS before reaching six months of age	4	(1.2)
By medical indication	3	(0.9)
Mastitis	2	(0.6)
Pain or cracked nipples	2	(0.6)
Grip problems	2	(0.6)
Mother’s use of medications	1	(0.3)
Mother’s illness	1	(0.3)
Other (low infant weight and mother’s mental health, travel, lack of milk bank, tiredness and lack of support, tongue tie diagnosis)	5	(1.5)

BF: Breastfeeding. EBF: Exclusive breastfeeding. HMS: Human milk substitute. The responses of three participants (0.9%) reported that the duration of their EBF was seven months. *^a^* Chi-square test: feeding with HMS during their hospital stay vs. other reasons, *p* = 0.07.

**Table 5:** Binary logistic regression model predicting the likelihood of not achieving exclusive breastfeeding for six months in Mexican mothers (*n* = 326, Mexico, 2022).

Factors Included in the Model	Variable	*n ^a^*	(%)	Bivariate Analysis *^b^*		Multivariate Analysis *^c^*		
				*p*	OR (CI 95%)	β	R^2^	*p*
In-hospital practices	24-h rooming-in							
	No	69	(21.0)	<0.001	13.16 (4.65–37.2)	−2.189	0.17	<0.001
	Yes	257	(79.0)					
	Start of BF within the first hour postpartum							
	No	150	(46.0)	<0.001	4.98 (2.99–8.31)	−1.280	0.26	<0.001
	Yes	176	(54.0)					
Gynecological	Number of children							
	One child	241	(73.9)	0.002	2.22 (1.34–3.68)	0.722	0.28	0.012
	More than one child	85	(26.1)					

BF: breastfeeding; OR: odds ratio; CI: confidence interval. β: regression coefficient (log-odds). *^a^* Responses from three participants (0.9%) reported that the duration of their EBF was seven months. *^b^* Bivariate analysis: Chi-square test and odds ratio. *^c^* Binary logistic regression; R^2^ Nagelkerke = 0.282. Cohen’s kappa of the model = 0.402. Note: Statistical significance was set at *p* < 0.05. Hosmer–Lemeshow goodness-of-fit test: Chi-square = 4.244, df = 4, *p* = 0.374, indicating a good model fit (*p* > 0.05).

## Data Availability

The data presented in this study are available on request from the corresponding author. The data are not publicly available due to privacy and ethical restrictions.

## Abbreviations

The following abbreviations are used in this manuscript:

BF	Breastfeeding
BFHI	Baby-Friendly Hospital Initiative
EBF	Exclusive breastfeeding
HM	Human Milk
HMS	Human Milk Substitutes
UNICEF	United Nations Children’s Fund
WHO	World Health Organization

## References

[1] World Health Organization Infant and Young Child Feeding. https://www.who.int/es/news-room/fact-sheets/detail/infant-and-young-child-feeding.

[2] Meek J.Y., Noble L. (2022). Technical Report: Breastfeeding and the Use of Human Milk. Pediatrics.

[3] González-Castell L.D., Unar-Munguía M., Bonvecchio-Arenas A., Ramírez-Silva I., Lozada-Tequeanes A.L. (2023). Prácticas de Lactancia Materna y Alimentación Complementaria En Menores de Dos Años de Edad En México. Salud. Publica Mex..

[4] Swigart T.M., Bonvecchio A., Théodore F.L., Zamudio-Haas S., Villanueva-Borbolla M.A., Thrasher J.F. (2017). Breastfeeding Practices, Beliefs, and Social Norms in Low-Resource Communities in Mexico: Insights for How to Improve Future Promotion Strategies. PLoS ONE.

[5] World Health Organization (1981). International Code of Marketing of Breast-Milk Substitutes.

[6] Crenshaw J.T., Budin W.D. (2020). Hospital Care Practices Associated With Exclusive Breastfeeding 3 and 6 Months After Discharge: A Multisite Study. J. Perinat. Educ..

[7] Volmanen P., Darnton-Hill I., Gonzales B. (1992). The Baby-Friendly Hospital Initiative. Asia Pac. J. Clin. Nutr..

[8] UNICEF IHAN: Lactancia Materna, Mejor Salud Para El Bebé—UNICEF. https://www.unicef.es/blog/lactancia/ihan-lactancia-materna-la-mejor-salud-para-el-bebe.

[9] Rollins N., Piwoz E., Baker P., Kingston G., Mabaso K.M., McCoy D., Ribeiro Neves P.A., Pérez-Escamilla R., Richter L., Russ K. (2023). Marketing of Commercial Milk Formula: A System to Capture Parents, Communities, Science, and Policy. Lancet.

[10] Pérez-Escamilla R., Martinez J.L., Segura-Pérez S. (2016). Impact of the Baby-friendly Hospital Initiative on Breastfeeding and Child Health Outcomes: A Systematic Review. Matern. Child Nutr..

[11] Li L., Song H., Zhang Y., Li H., Li M., Jiang H., Yang Y., Wu Y., Gu C., Yu Y. (2021). Public Health. Int. J. Environ. Res. Public Health.

[12] McFadden A., Gavine A., Renfrew M.J., Wade A., Buchanan P., Taylor J.L., Veitch E., Rennie A.M., Crowther S.A., Neiman S. (2017). Support for Healthy Breastfeeding Mothers with Healthy Term Babies. Cochrane Database Syst. Rev..

[13] Spaeth A., Zemp E., Merten S., Dratva J. (2018). Baby-Friendly Hospital Designation Has a Sustained Impact on Continued Breastfeeding. Matern. Child Nutr..

[14] World Health Organization, United Nations Children’s Fund (2018). Protecting, Promoting and Supporting Breastfeeding in Facilities Providing Maternity and Newborn Services: Implementing the Revised Baby-Friendly Hospital Initiative 2018.

[15] Prian-Gaudiano A., Horta-Carpinteyro D., Sarmiento-Aguilar A. (2024). Relationship between Skin-to-Skin Contact during the First Hour of Life and Duration of Exclusive Breastfeeding. Bol. Med. Hosp. Infant. Mex..

[16] IHAN, UNICEF Pasos Para Ser IHAN—Hospitales. https://www.ihan.es/acreditacion-hospitales/.

[17] Nicholson W.K., Silverstein M., Wong J.B., Chelmow D., Coker T.R., Davis E.M., Fernandez A., Gibson E., Jaén C.R., Krousel-Wood M. (2025). Primary Care Behavioral Counseling Interventions to Support Breastfeeding. JAMA.

[18] Dean A.G., Sullivan K.M., Soe M.M. OpenEpi: Open Source Epidemiologic Statistic for Public Health. www.OpenEpi.com.

[19] Centers for Disease Control and Prevention Epi Info. https://www.cdc.gov/epiinfo/index.html.

[20] Organización Mundial de la Salud (2010). La Alimentación Del Lactante y Del Niño Pequeño: Capítulo Modelo Para Libros de Texto Dirigidos a Estudiantes de Medicina y Otras Ciencias de La Salud.

[21] Vásquez-Garibay E.M. (2016). Primer Año de Vida. Leche Humana y Sucedáneos de La Leche Humana. Gac. Med. Mex..

[22] del Mazo-Tomé P.L., Suárez-Rodríguez M. (2019). Prevalencia de La Alimentación Exclusiva Con Lactancia Materna En Recién Nacidos Sanos. Bol. Med. Hosp. Infant. Mex..

[23] Ibarra-Ortega A., Vásquez-Garibay E.M., Larrosa-Haro A., Castro-Albarrán J., Vizmanos-Lamotte B. (2020). Using a Lactation Room at the Workplace Is Associated with Longer Breastfeeding Duration in Working Mothers. Nutr. Hosp..

[24] Asociación Mexicana de Agencias de Inteligencia de Mercado y Opinión AMAI Nivel Socio Económico NSE. https://www.amai.org/NSE/index.php.

[25] Primera Reunión de Consenso Académico en Medicina Familiar de Organismos e Instituciones Educativas y de Salud (2005). Elementos Esenciales de La Medicina Familiar. Conceptos Básicos Para El Estudio de Las Familias. Código de Bioética En Medicina Familiar.

[26] Instituto Nacional de Estadística y Geografía (INEGI), Instituto Nacional de Salud Pública (INSP) (2019). Encuesta Nacional de Salud y Nutrición 2018—ENSANUT: Diseño Conceptual.

[27] Hörnell A., Hofvander Y., Kylberg E. (2001). Solids and Formula: Association with Pattern and Duration of Breastfeeding. Pediatrics.

[28] Chimoriya R., Scott J.A., John J.R., Bhole S., Hayen A., Kolt G.S., Arora A. (2020). Determinants of Full Breastfeeding at 6 Months and Any Breastfeeding at 12 and 24 Months among Women in Sydney: Findings from the HSHK Birth Cohort Study. Int. J. Environ. Res. Public Health.

[29] Lechosa-Muñiz C., Paz-Zulueta M., Sota S.M., de Adana Herrero M.S., del Rio E.C., Llorca J., Cabero-Perez M.J. (2020). Factors Associated with Duration of Breastfeeding in Spain: A Cohort Study. Int. Breastfeed. J..

[30] Santana G.S., Giugliani E.R.J., de Vieira T.O., Vieira G.O. (2018). Factors Associated with Breastfeeding Maintenance for 12 Months or More: A Systematic Review. J. Pediatr. (Rio. J.).

[31] Valenzuela Galleguillos S., Vásquez Pinto E., Gálvez Ortega P. (2016). Factores Que Influyen En La Disminución de Lactancia Materna Exclusiva Hasta Los 6 Meses de Vida: Revisión Sistemática y Contexto En Chile. Rev. Int. Salud Materno. Fetal..

[32] Ramiro González M.D., Ortiz Marrón H., Arana Cañedo-Argüelles C., Esparza Olcina M.J., Cortés Rico O., Terol Claramonte M., Ordobás Gavín M. (2018). Prevalencia de La Lactancia Materna y Factores Asociados Con El Inicio y La Duración de La Lactancia Materna Exclusiva En La Comunidad de Madrid Entre Los Participantes En El Estudio ELOIN. Pediatr. (Engl. Ed.).

[33] Ávila-Ortiz M.N., Castro-Sánchez A.E., Martínez-González E.A., Núñez-Rocha G.M., Zambrano-Moreno A. (2020). Factors Associated with Abandoning Exclusive Breastfeeding in Mexican Mothers at Two Private Hospitals. Int. Breastfeed. J..

[34] Sámano R., Chico-Barba G., Armenteros-Martínez T., Escamilla-Fonseca N., Piélago-Álvarez C., Aguilar-Álvarez J., Méndez-Celayo S. (2018). Barreras y Facilitadores Para La Práctica de Lactancia Materna Exclusiva En Un Grupo de Madres de La Ciudad de México. Arch. Latinoam Nutr..

[35] Garfias-Barrera A., Márquez-Cardoso E., Moreno-Aguilera F., Bazán-Castro M. (2007). Factores de Riesgo Maternos y Familiares Que Influyen En El Abandono de La Lactancia Materna. Rev. De Espec. Médico-Quirúrgicas.

[36] Niño R., Gioconda S.E., Atalah E. (2012). Factores Asociados a La Lactancia Materna Exclusiva. Rev. Chil. Pediatr..

[37] Unar-Munguía M., Lozada-Tequeanes A.L., González-Castell D., Cervantes-Armenta M.A., Bonvecchio A. (2021). Breastfeeding Practices in Mexico: Results from the National Demographic Dynamic Survey 2006–2018. Matern. Child Nutr..

[38] Buckman C., Diaz A.L., Tumin D., Bear K. (2020). Parity and the Association Between Maternal Sociodemographic Characteristics and Breastfeeding. Breastfeed. Med..

[39] Vural F., Vural B. (2017). The Effect of Prenatal and Postnatal Education on Exclusive Breastfeeding Rates. Minerva Pediatr..

[40] Wagner S., Kersuzan C., Gojard S., Tichit C., Nicklaus S., Thierry X., Charles M.A., Lioret S., de Lauzon-Guillain B. (2019). Breastfeeding Initiation and Duration in France: The Importance of Intergenerational and Previous Maternal Breastfeeding Experiences—Results from the Nationwide ELFE Study. Midwifery.

[41] McCoy M.B., Heggie P. (2020). In-Hospital Formula Feeding and Breastfeeding Duration. Pediatrics.

[42] Zarshenas M., Zhao Y., Scott J.A., Binns C.W. (2020). Determinants of Breastfeeding Duration in Shiraz, Southwest Iran. Int. J. Environ. Res. Public Health.

[43] Sampieri C.L., Fragoso K.G., Córdoba-Suárez D., Zenteno-Cuevas R., Montero H. (2022). Influence of Skin-to-Skin Contact on Breastfeeding: Results of the Mexican National Survey of Demographic Dynamics, 2018. Int. Breastfeed. J..

[44] Vila-Candel R., Duke K., Soriano-Vidal F.J., Castro-Sánchez E. (2018). Affect of Early Skin-to-Skin Mother–Infant Contact in the Maintenance of Exclusive Breastfeeding: Experience in a Health Department in Spain. J. Hum. Lact..

[45] Castillo-Cruz R.A., Iracheta-Gerez M.d.l.L., Macias-Parra M., Esparza-Aguilar M. (2022). Factors Associated with the Duration of Breastfeeding: The Practices of Mexican Mothers in a Megacity and in the Agricultural Town. Int. J. Environ. Res. Public Health.

[46] Daniels F., Sawangkum A., Kumar A., Coombs K., Louis-Jacques A., Ho T.T.B. (2023). Skin to Skin Contact Correlated with Improved Production and Consumption of Mother’s Own Milk. Breastfeed. Med..

[47] Pérez-Escamilla R., Tomori C., Hernández-Cordero S., Baker P., Barros A.J.D., Bégin F., Chapman D.J., Grummer-Strawn L.M., McCoy D., Menon P. (2023). Breastfeeding: Crucially Important, but Increasingly Challenged in a Market-Driven World. Lancet.

[48] Wu H.-L., Lu D.-F., Tsay P.-K. (2022). Rooming-In and Breastfeeding Duration in First-Time Mothers in a Modern Postpartum Care Center. Int. J. Environ. Res. Public Health.

[49] Habte M.B., Abdulahi M., Plusquin M., Cosemans C. (2025). Effectiveness of Baby-Friendly Hospital Initiative on Early Initiation and Exclusive Breastfeeding Practice: Systematic Review and Meta-Analysis. Nutrients.

